# Vinculin-cell membrane interactions

**DOI:** 10.18632/oncotarget.5868

**Published:** 2015-09-28

**Authors:** David T. Brown, Tina Izard

**Affiliations:** Cell Adhesion Laboratory, Department of Cancer Biology and Department of Immunology and Microbial Sciences, The Scripps Research Institute, Jupiter, FL, USA

**Keywords:** cytoskeleton, focal adhesions, lipids, phosphatidylinositide 4,5-bisphosphate (PIP2), vinculin

Focal adhesions (FAs) are macromolecular complexes that connect the actin cytoskeleton to the extracellular matrix (ECM). These cell junctions are highly spatially organized yet dynamic organelles that transduce force from the actin cytoskeleton to transmembrane proteins known as integrins, which are heterodimers that bind to ECM components such as fibronectin, collagen, and laminin. The intracellular tails of integrin connect indirectly to the actin network *via* interactions with core FA proteins such as talin and α-actinin. Force is transmitted from F-actin to integrins *via* these core components to promote cell migration by a mechanism that has been termed a “molecular clutch” [1].

Vinculin is an essential protein component of FAs thought to stabilize FAs and to regulate force transmission. *Vinculin* null mice die during embryogenesis and embryonic fibroblasts derived from these mice display marked defects in cell movement. Vinculin is a 1066 residue protein consisting of a 91 kDa head domain (VH) and a 21 kDa five-helix bundle tail domain (Vt) separated by a proline-rich linker. Cytoplasmic vinculin is kept in a closed, auto-inhibited conformation by hydrophobic interactions between VH and Vt. Activation of vinculin in FAs uncovers binding sites for numerous FA proteins including talin, α-actinin, α- and β-catenin, and E-cadherin in VH, and paxillin, F-actin, and raver1 in Vt. Activated vinculin is also able to bind membrane-bound lipids such as phosphatidylinositide 4,5-bisphosphate (PIP_2_). Inositol phospholipids are components of numerous signaling pathways but the function of PIP_2_ binding by vinculin is unclear.

We recently reported the crystal structure of human vinculin bound to PIP_2_, which revealed that PIP_2_ binding alters vinculin structure to direct oligomerization and that simultaneous binding of PIP_2_ and F-actin is structurally possible [2]. The structure also unequivocally identified the vinculin residues involved in PIP_2_ binding thereby allowing the design of mutant constructs specifically deficient in PIP_2_ binding. These observations should be interpreted in light of recent reports describing the nanoscale structure of FAs derived from super-resolution microscopy [1]. These studies indicated that FAs are stratified vertically into three layers: a membrane-proximal integrin signaling layer (ISL), an actin regulatory layer (ARL) located ~60 nm higher, and an intermingled force transducing layer (FTL) between the ISL and ARL. Because of the distance between the ISL and the ARL it was suggested that only talin could simultaneously engage integrins and actin. Vinculin is distributed between all three of the axial FA layers but the distance between the ISL and the ARL would preclude a single vinculin molecule from binding simultaneously with PIP_2_ in the ISL and F-actin in the ARL. Thus, it was suggested that vinculin “climbs” talin like a ladder to reach the ARL and bind F-actin. However, the data from super-resolution microscopy do not provide direct information on the dynamic behavior of proteins at the nanoscale level. Further, many of the proteins were enriched in a particular layer but also rather broadly distributed consistent with these proteins moving between layers as FAs mature or mediate translocation.

*Vinculin* null (−/−) mouse fibroblasts form small and ineffective FAs and display defects in cell spreading and wound healing assays. We and others have shown that these phenotypes can be rescued by the expression of GFP-tagged wild-type vinculin [2, 3]. Based on the PIP_2_-bound vinculin structure we designed GFP-vinculin constructs containing specific mutants designed to prevent PIP_2_ binding without compromising other vinculin functions. When expressed in *vinculin* (−/−) cells, these lipid-binding defective mutants were actually recruited to FAs more effectively than wild type GFP-vinculin (Brown & Izard, unpublished observation) but did not rescue the defects in cell spreading or wound healing. Further, FRAP analysis revealed that while the entire population of wild type GFP turned over in less than a minute, as much as 90% of the lipid binding defective GFP-vinculin was statically bound. We conclude that PIP_2_ binding is essential for dynamic vinculin interactions with FAs, which is crucial for normal FA function.

It was previously suggested that lipid binding might serve to release rather than recruit vinculin to FAs [4]. Regulation of this process in response to physiological cues might represent a means to modulate cell movements and several recent observations support such a view. FAs are polarized structures and FRAP studies showed distinct differences in vinculin exchange rates at the distal and proximal tips [5]. The FA residence time of full-length vinculin but not VH correlates with applied force [6]. Finally, it was recently reported that ECM stiffness, such as that found at the invasive border of breast tumors, stabilizes vinculin/talin/actin interactions, which facilitates PIP_2_ conversion to PIP_3_ and the activation of signaling pathways that might contribute to cancer cell invasion [7]. Clearly, we are a long way from understanding the details of the connection between phosphoinositide signaling and FA-mediated cell migrations but they are likely to have significant functional implications for normal cell function and tumor cell progression and metastasis.
